# The Finnish Diabetes Risk Score (FINDRISC) Combined with Explainable Machine Learning for Detection of Previously Unrecognized Dysglycemia in Primary Care: A CatBoost-Based Approach

**DOI:** 10.3390/healthcare14193214

**Published:** 2026-09-29

**Authors:** Fevziye Türkoğlu Genç, Mehmet Yildiz

**Affiliations:** 1 Department of Internal Medicine, Kanuni Sultan Süleyman Training and Research Hospital, İstanbul 34303, Türkiye; fevziyeturkoglu@gmail.com; 2 Giresun Provincial Health Directorate, Bulancak Sehit Er Enver Erdogan Family Health Center, Giresun 28300, Türkiye

**Keywords:** prediabetes, type 2 diabetes mellitus, FINDRISC, CatBoost, machine learning, explainable artificial intelligence, SHAP, primary care

## Abstract

**Highlights:**

**What are the main findings?**

- FINDRISC was the dominant discriminator of previously unrecognized dysglycemia, retaining strong discrimination within a CatBoost-based framework.
- Adding SHAP-selected routine clinical and laboratory variables produced only minimal incremental discrimination over FINDRISC alone on the held-out test set (AUC 0.840 vs. 0.835; ΔAUC = 0.005, 95% bootstrap CI −0.012 to 0.022).

**What are the implications of the main findings?**

- FINDRISC can be a useful and practical case-finding tool in primary care and internal medicine; in this dataset, most discrimination was already captured by the score itself.
- Explainable machine learning can characterize contributions from additional routine variables, but the present internally evaluated results do not establish clinically meaningful incremental benefit over FINDRISC alone.

**Abstract:**

**Background/Objectives:** To evaluate a CatBoost-based machine learning (ML) approach for detecting previously unrecognized dysglycemia at the index primary-care visit and to assess the contribution of the Finnish Diabetes Risk Score (FINDRISC) and routinely available demographic, clinical, and laboratory variables using SHAP. **Methods:** This retrospective cross-sectional study included 2033 adults without previously diagnosed prediabetes or diabetes. The dataset was randomly split once into a training set of 1626 participants and an internal held-out test set of 407 participants. Three CatBoost feature-set configurations were evaluated: FINDRISC Only, SHAP-selected variables Without FINDRISC, and the same variables With FINDRISC. Five-fold stratified cross-validation and hyperparameter optimization were performed within the training set; the held-out test set was not used for model fitting or tuning. Raw FINDRISC was additionally evaluated as a non-ML benchmark. Test-set evaluation included discrimination, classification metrics, calibration, and paired-bootstrap comparison of AUCs. **Results:** Overall, 935 (46.0%) participants had screen-detected dysglycemia. On the held-out test set, AUCs were 0.758 for Without FINDRISC, 0.835 for FINDRISC Only, 0.840 for With FINDRISC, and 0.833 for raw FINDRISC. The incremental AUC of With FINDRISC versus FINDRISC Only was 0.005 (95% paired bootstrap CI −0.012 to 0.022; *p* = 0.556), indicating no statistically significant improvement. Brier scores were 0.203, 0.169, and 0.166 for Without FINDRISC, FINDRISC Only, and With FINDRISC, respectively. **Conclusions:** FINDRISC accounted for most of the observed discrimination for prevalent, previously unrecognized dysglycemia. Adding routine clinical and laboratory variables within CatBoost produced only a small, statistically non-significant incremental improvement. These findings should be regarded as preliminary internal evidence and require prospective external validation.

## 1. Introduction

According to the International Diabetes Federation (IDF), approximately 589 million adults aged 20–79 years were living with diabetes worldwide in 2024. The IDF reported a global diabetes prevalence of 11.11% among adults and projected that the number of individuals with diabetes will reach approximately 853 million by 2050 [1]. A major challenge in diabetes is its delayed diagnosis. Approximately 45% of adults with diabetes are unaware of their condition, indicating that nearly 240 million people worldwide remain undiagnosed [2]. Delayed diagnosis may prevent the recognition of diabetes before diabetes-related complications develop [3]. Intensive lifestyle interventions in individuals with impaired glucose tolerance have been shown to reduce the risk of developing type 2 diabetes mellitus (T2DM) by approximately 58% [4]. Early diagnosis is also crucial for preserving pancreatic β-cell function. Impairment of β-cell function begins during the prediabetes stage and progressively worsens with disease progression; therefore, interventions implemented at an early stage may contribute to preserving β-cell function [5].

The Finnish Diabetes Risk Score (FINDRISC) was developed by Lindström and Tuomilehto in 2003 as an eight-item non-invasive risk assessment tool that includes age, body mass index, waist circumference, physical activity, dietary habits, antihypertensive medication use, history of elevated blood glucose, and family history of diabetes. FINDRISC is calculated on a scale ranging from 0 to 26 points, and higher scores are directly associated with an increased risk of developing T2DM within the following 10 years [6].

In addition to classical risk factors, biochemical and hematological parameters obtained during routine clinical practice also play an important role in the development of T2DM. Hematological and biochemical parameters such as triglycerides, cholesterol, alanine aminotransferase (ALT), gamma-glutamyl transferase (GGT), uric acid, white blood cell (WBC) count, and monocyte count (Mon#) are associated with insulin resistance, metabolic disorders, and the development of T2DM, whereas systolic blood pressure, serum iron level, urea, and comorbidity burden may also contribute to the assessment of individual T2DM risk [7,8,9].

Advances in artificial intelligence (AI) have enabled the widespread application of machine learning (ML) algorithms in various fields. CatBoost, a gradient boosting-based ML algorithm, differs from other gradient boosting algorithms by processing categorical variables using its own encoding approach and reducing prediction shift through the ordered boosting method [10]. SHapley Additive exPlanations (SHAP) is a model interpretation method based on Shapley values derived from game theory that enables the contribution of each feature to model predictions to be explained at both the individual and global levels. SHAP is an important method for explainable artificial intelligence (XAI) [11].

The aim of this study was to evaluate a CatBoost-based ML framework using FINDRISC together with routine biochemical and clinical parameters obtained at the index visit to detect previously unrecognized dysglycemia (prediabetes or diabetes-range glycemia) in adults without a previous diagnosis of prediabetes or diabetes. SHAP analysis was used to quantify feature contributions and improve interpretability. We further compared three CatBoost feature-set configurations and the raw FINDRISC to determine whether routinely available variables provided incremental discrimination beyond FINDRISC.

## 2. Materials and Methods

### 2.1. Study Design and Participants

We conducted a retrospective cross-sectional study using electronic medical records obtained from a primary care family health center. The study population consisted of individuals registered at the family health center. We included adults aged ≥18 years who attended the center between 1 March 2025 and 1 March 2026, and had no previous diagnosis of prediabetes or diabetes. Of 9874 individuals registered at the family health center, 2033 were included in the final analysis after applying the eligibility criteria and excluding individuals with missing required data. We calculated total disease count as the number of recorded chronic disease categories for each participant (Figure 1). The count included chronic obstructive pulmonary disease, hyperlipidemia, coronary artery disease, psychiatric disorders, cardiac arrhythmias, cerebrovascular disease, epilepsy, chronic kidney disease, and heart failure.

The study protocol was approved by the Giresun University Medical Research Ethics Committee (Date: 11 June 2026; Decision No: 11.06.2026/09). We conducted the study in accordance with the principles of the Declaration of Helsinki. Because this retrospective study used only anonymized electronic medical records and involved neither direct patient contact nor any intervention, the ethics committee waived the requirement for written informed consent. We de-identified all data before statistical analysis to protect participant confidentiality.

### 2.2. Outcome Definition

We classified glycemic status according to the 2026 American Diabetes Association (ADA) Standards of Care [12]. Prediabetes-range glycemia was defined as FPG ≥ 100 and <126 mg/dL or HbA1c ≥ 5.7% and <6.5%, and diabetes-range glycemia as FPG ≥ 126 mg/dL or HbA1c ≥ 6.5%. Because glycemic status was determined from measurements available at the index visit and confirmatory repeat testing was not available, we used the terms screen-detected diabetes-range glycemia and previously unrecognized dysglycemia rather than confirmed incident T2DM. Participants meeting either prediabetes-range or diabetes-range criteria formed the positive combined case-finding endpoint; those with normal glycemia formed the negative group. The combined endpoint was chosen to reflect a primary-care case-finding approach in which both categories warrant early recognition, close follow-up, and, when appropriate, referral to an internal medicine specialist for further evaluation and management.

### 2.3. Data Preprocessing

Variable names were standardized, a unique patient identifier was generated, and categorical variables were coded for analysis. Continuous variables were retained on their original scale. Variables with fewer than four unique values were treated as categorical for CatBoost because these variables represented discrete/binary clinical attributes rather than continuously measured quantities.

### 2.4. Model Development

CatBoost is the prespecified ML algorithm; the study compared feature-set configurations rather than different ML algorithms. The complete dataset was randomly partitioned using the first fold generated by a five-fold KFold procedure with shuffling and a random state of 3, yielding a model-development set of 1626 participants and an internal held-out test set of 407 participants. This initial development/test partition was not outcome-stratified. The held-out test set was not used for feature assessment, hyperparameter optimization, or model fitting.

Model development was performed within the training set using five-fold stratified cross-validation. We evaluated 432 hyperparameter combinations across iterations (100, 200, 300, 400, 500, 600, 700, 800, and 1000), tree depth (1, 2, 3, and 4), learning rate (0.005, 0.01, 0.05, and 0.1), and L2 leaf regularization (2, 7, and 10). The selected CatBoost configuration used 100 iterations, a tree depth of 3, a learning rate of 0.1, and an L2 leaf regularization value of 10. We ranked the hyperparameter combinations primarily using the product of accuracy, AUC, and sensitivity, with AUC, sensitivity, F1-score, specificity, and accuracy used as subsequent ranking criteria. Balanced class weights were retained in the original model-development pipeline. All final performance estimates reported for the held-out test set were calculated only after model development was complete.

We used SHAP within the model-development set to quantify global feature contributions. We evaluated feature importance using five-fold KFold cross-validation. In each fold, we calculated SHAP values using the fold-specific training data. We defined feature importance as the mean absolute SHAP value for each variable. The number of variables to be retained was not fixed in advance. A SHAP importance threshold of >0.015 was applied at the fold level, and a variable was retained in the final feature set if its mean absolute SHAP importance exceeded this threshold in at least one of the five development folds. Fifteen non-FINDRISC variables met this criterion. These variables included age, ALT, GGT, WBC, monocyte count, uric acid, triglyceride, cholesterol, systolic blood pressure, iron, urea, creatinine, sex, total disease count, and lactate dehydrogenase (LDH). We combined the fold-level SHAP results to define the final feature set. We did not repeat feature selection independently within each subsequent model-evaluation fold. We treated FINDRISC as a single total-score feature. We evaluated three CatBoost configurations. The FINDRISC Only model used the total FINDRISC as the sole input. The Without FINDRISC model used the 15 selected variables. The With FINDRISC model used the same 15 variables together with the total FINDRISC. We did not repeat SHAP-based feature selection after removing FINDRISC. We also evaluated the raw FINDRISC without ML transformation as a conventional reference benchmark.

### 2.5. Model Performance Evaluation

Model performance was assessed using AUC, sensitivity, specificity, precision, accuracy, weighted F1-score, average precision (AP), and Matthews correlation coefficient (MCC). Binary classification metrics used the prespecified probability threshold of 0.50; the held-out test set was not used to optimize this threshold. Test-set AUC confidence intervals were estimated by bootstrap resampling. To quantify incremental discrimination, the AUC difference (ΔAUC) between the With FINDRISC and FINDRISC Only configurations was evaluated using 10,000 paired, outcome-stratified bootstrap resamples of the same held-out participants, with a two-sided bootstrap *p* value and percentile 95% confidence interval. We assessed calibration on the held-out test set using the Brier score, calibration intercept, and calibration slope. Raw FINDRISC discrimination was evaluated by ROC analysis without fitting an ML model. Continuous variables are presented as mean ± SD and categorical variables as n (%).

### 2.6. Software

All analyses were performed using Python 3.14.3, pandas 3.0 and NumPy 2.3 were used for data management; CatBoost 1.2.10 for model development; SHAP 0.2.0 for model interpretation; scikit-learn 1.9.1 for data splitting, cross-validation, hyperparameter optimization, discrimination, classification, calibration, and resampling-based evaluation; and Matplotlib 3.11.2, Seaborn 0.13.2, and Plotly 7.1.0 for visualization. The results were reported against the TRIPOD+AI framework (Supplementary Material File S1) [13].

### 2.7. Use of Generative Artificial Intelligence

A generative artificial intelligence tool (ChatGPT, GPT-5.6 Sol, OpenAI, San Francisco, CA, USA) was used during manuscript preparation to assist with language refinement and the drafting and revision of selected textual content. It was not used for study design, data collection, data generation, or statistical analysis. All AI-assisted content was critically reviewed, verified, and edited by the authors, who take full responsibility for the final manuscript.

## 3. Results

A total of 2033 individuals were included in the study. The mean age was 46.9 ± 16.3 years; 1095 (53.9%) were female and 938 (46.1%) were male. Overall, 1098 (54.0%) participants had normal glycemia, 818 (40.2%) had prediabetes-range glycemia, and 117 (5.8%) had diabetes-range glycemia. The combined dysglycemia endpoint included 935 participants (46.0%). Mean FINDRISC was 8.60 ± 5.15 in the negative group and 16.01 ± 5.26 in the positive group. Baseline demographic, clinical, and laboratory characteristics stratified by outcome are summarized in Table 1.

SHAP-based feature selection retained 15 non-FINDRISC variables. The Without FINDRISC feature set comprised age, ALT, GGT, WBC, monocyte count, uric acid, triglyceride, cholesterol, systolic blood pressure, iron, urea, creatinine, sex, total disease count, and LDH. In the With FINDRISC configuration, the total FINDRISC was added as a sixteenth feature and showed the largest global SHAP contribution (Figure 2).

The random, non-stratified hold-out split yielded a model-development set of 1626 participants, including 753 positive and 873 negative cases, and an internal held-out test set of 407 participants, including 182 positive and 225 negative cases. Five-fold stratified cross-validation was subsequently performed only within the model-development set. The held-out test set remained excluded from feature assessment, hyperparameter optimization, and model fitting.

During five-fold cross-validation, the With FINDRISC configuration showed the highest discriminative performance, with an AUC of 0.86. The AUC was 0.84 for the FINDRISC Only configuration and 0.78 for the Without FINDRISC configuration. These cross-validation estimates were used to assess model performance during the development stage, whereas the final comparisons between the three CatBoost configurations were based on the locked held-out test set (Table 2).

On the held-out test set, the Without FINDRISC configuration had an AUC of 0.758, a sensitivity of 0.76, a specificity of 0.60, and an MCC of 0.369. The FINDRISC Only configuration had an AUC of 0.835, a sensitivity of 0.78, a specificity of 0.75, and an MCC of 0.524. The With FINDRISC configuration had an AUC of 0.840, a sensitivity of 0.80, a specificity of 0.73, and an MCC of 0.527 (Table 3, Figure 3). At the prespecified classification threshold of 0.50, the With FINDRISC configuration yielded 165 true negatives, 60 false positives, 37 false negatives, and 145 true positives (Table 4).

When evaluated directly without machine learning, the raw FINDRISC had an AUC of 0.833. The difference in AUC between the With FINDRISC and FINDRISC Only configurations was 0.005 (95% CI [−0.012, 0.022]; *p* = 0.556).

Calibration was assessed on the held-out test set using Brier scores, calibration intercepts, and calibration slopes. The Without FINDRISC configuration had a Brier score of 0.203, a calibration intercept of −0.274, and a calibration slope of 0.926. The FINDRISC Only configuration had a Brier score of 0.169, a calibration intercept of −0.342, and a calibration slope of 0.913. The With FINDRISC configuration had a Brier score of 0.166, a calibration intercept of −0.376, and a calibration slope of 0.974.

## 4. Discussion

In this retrospective cross-sectional case-finding study, FINDRISC accounted for most of the discrimination for previously unrecognized dysglycemia. The With FINDRISC CatBoost configuration achieved an AUC of 0.840, compared with 0.835 for FINDRISC Only and 0.833 for the raw FINDRISC. The incremental difference between With FINDRISC and FINDRISC Only was only 0.005 and was not statistically significant on paired bootstrap analysis. Thus, a numerically higher AUC should not be interpreted as evidence of a clinically meaningful improvement. In contrast, the Without FINDRISC configuration had substantially lower discrimination (AUC 0.758), reinforcing that the total FINDRISC carried most of the useful discriminatory information in this study.

A systematic review and meta-analysis including 25 cross-sectional and 7 cohort studies reported that the AUC of FINDRISC ranged from 0.694 to 0.755 across cut-off values between 9 and 15, with the best screening performance observed at cut-off values of 9 and 10 [14]. A large European validation study involving 11,444 adults from primary health care centres reported an optimal FINDRISC cut-off of 14 points for detecting both diabetes and glucose impairment in community screening, with an associated AUC of 0.76 [15]. A Korean study comparing non-laboratory and laboratory diabetes prediction models reported that non-laboratory risk scores achieved sensitivity values of up to 81%, specificity values of up to 58%, and AUC values ranging from 0.70 to 0.78, whereas a laboratory model based on fasting plasma glucose and HbA1c achieved an AUC of 0.84. Adding the non-laboratory risk score to the laboratory model increased the AUC to 0.85 [16]. Collectively, these studies indicate that diabetes prediction models incorporating glycemic markers, such as fasting plasma glucose or HbA1c, achieve higher discriminative performance, with reported AUC values of approximately 0.84–0.85. In contrast, non-laboratory models based solely on demographic, anthropometric, and lifestyle-related variables generally report AUC values ranging from 0.70 to 0.78.

A Korean study developed and compared five ML algorithms using demographic, anthropometric, lifestyle, and clinical variables without incorporating fasting plasma glucose or HbA1c. LightGBM achieved the highest predictive performance, with an AUC of 0.82, a sensitivity of 84.8%, and a specificity of 64.8% [17]. In addition to achieving high discriminative performance, selecting a cut-off that provides a balanced trade-off between sensitivity and specificity has been recommended to improve the overall diagnostic utility of prediction models [18]. Although this model demonstrated high discriminative performance, the difference between sensitivity and specificity was greater than that observed in our study. In contrast, our CatBoost model achieved a more balanced sensitivity and specificity while maintaining high discriminative performance.

A recent study comparing XGBoost and CatBoost reported that XGBoost achieved the highest predictive performance with an AUC of 99.29%, an accuracy of 96.07%, and an F1-score of 96.10%, whereas CatBoost achieved an AUC of 99.01% and an accuracy of 95.31% [19]. However, FPG was the most influential predictor in that model, accounting for 41% of the overall feature importance. In contrast, we intentionally excluded FPG and HbA1c from the predictor set because these variables were used to define the study outcome of prediabetes-range or diabetes-range glycemia according to the ADA criteria [12]. Including these diagnostic biomarkers as input features could introduce information leakage and artificially inflate the model performance.

The raw FINDRISC showed good discriminative performance in the present study, with an AUC of 0.833, indicating that the score alone effectively distinguished participants with and without dysglycemia. This may partly reflect the spectrum of the present primary-care study population, in which 46% met the combined dysglycemia endpoint, and the fact that FINDRISC includes a history of elevated blood glucose. These characteristics may enrich the sample for metabolic risk and should be considered when interpreting transportability to unselected primary-care populations.

The SHAP findings should also be interpreted in light of predictor overlap. FINDRISC already incorporates age, BMI, waist circumference, physical activity, dietary habits, antihypertensive treatment, previous elevated glucose, and family history. Therefore, adding correlated or conceptually overlapping clinical variables can redistribute SHAP attribution across features. In the present model, FINDRISC remained the dominant global contributor, while directions and magnitudes of laboratory-feature contributions should be viewed as model-specific associations rather than causal effects.

The combined model therefore showed the lowest Brier score and a calibration slope closest to 1, although external calibration remains unknown. Because balanced class weights were used in the original training pipeline, calibration findings are reported explicitly rather than assuming that good discrimination implies well-calibrated probabilities.

Future work should prioritize prospective external validation in independent, multicenter, and geographically diverse primary-care or internal medicine populations; comparison with established diabetes risk scores and conventional reference approaches such as logistic regression; evaluation of the individual FINDRISC components where available; and assessment of clinical utility using prespecified screening thresholds and decision-curve analysis.

### Limitations

This study has several limitations. First, its retrospective cross-sectional design identifies prevalent, previously unrecognized dysglycemia at the index visit and does not establish future risk, temporality, or causality. Second, diabetes-range glycemia was defined from a single FPG or HbA1c measurement without confirmatory repeat testing; therefore, these findings should not be interpreted as confirmed incident T2DM diagnoses. Third, prediabetes-range and diabetes-range glycemia were combined into one case-finding endpoint; although this reflects a pragmatic screening objective, the two states have different clinical implications. Fourth, this was a single-center study with internal hold-out evaluation only and therefore does not establish transportability. Fifth, the positive outcome prevalence of 46% suggests a risk-enriched spectrum relative to general population screening and may limit generalizability. The exclusion of individuals with missing required data may have introduced selection bias. Sixth, FINDRISC contains information that overlaps conceptually with several clinical predictors, which can affect SHAP attribution among correlated inputs. Finally, the study evaluated CatBoost feature-set configurations rather than benchmarking multiple ML algorithms; comparisons with logistic regression and other ML methods, as well as models using the eight FINDRISC components separately, require future datasets and external validation.

## 5. Conclusions

In this internally evaluated retrospective study, FINDRISC provided most of the discrimination for detecting previously unrecognized dysglycemia in primary care. CatBoost using FINDRISC alone performed almost identically to the raw FINDRISC, and adding selected routine clinical and laboratory variables increased AUC by only 0.005, with a 95% paired-bootstrap confidence interval crossing zero. The principal practical implication is therefore not that ML meaningfully improves FINDRISC, but that FINDRISC remains a strong, simple case-finding instrument in this study population while explainable ML can describe additional feature contributions. A single parameter such as FINDRISC can be a practical and accessible marker for predicting prediabetes and diabetes. These findings may also be relevant to internal medicine practice, where simple case-finding tools can support the identification of individuals who warrant further glycemic assessment. Prospective, multicenter external validation and formal clinical-utility assessment are required before any ML-enhanced approach can be recommended for routine screening.

## Figures and Tables

**Figure 1:**
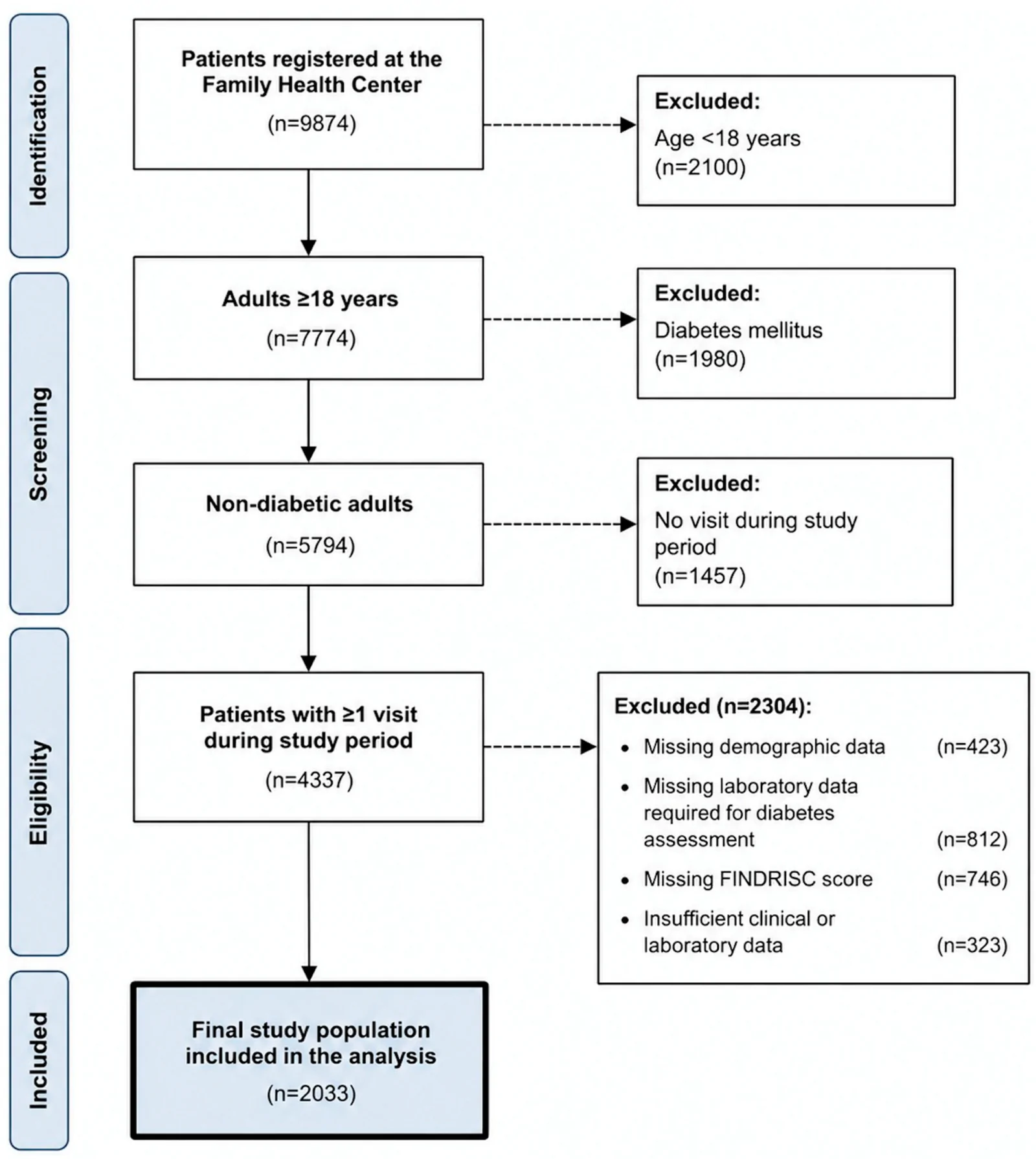
Flowchart of the selection process of the study population.

**Figure 2:**
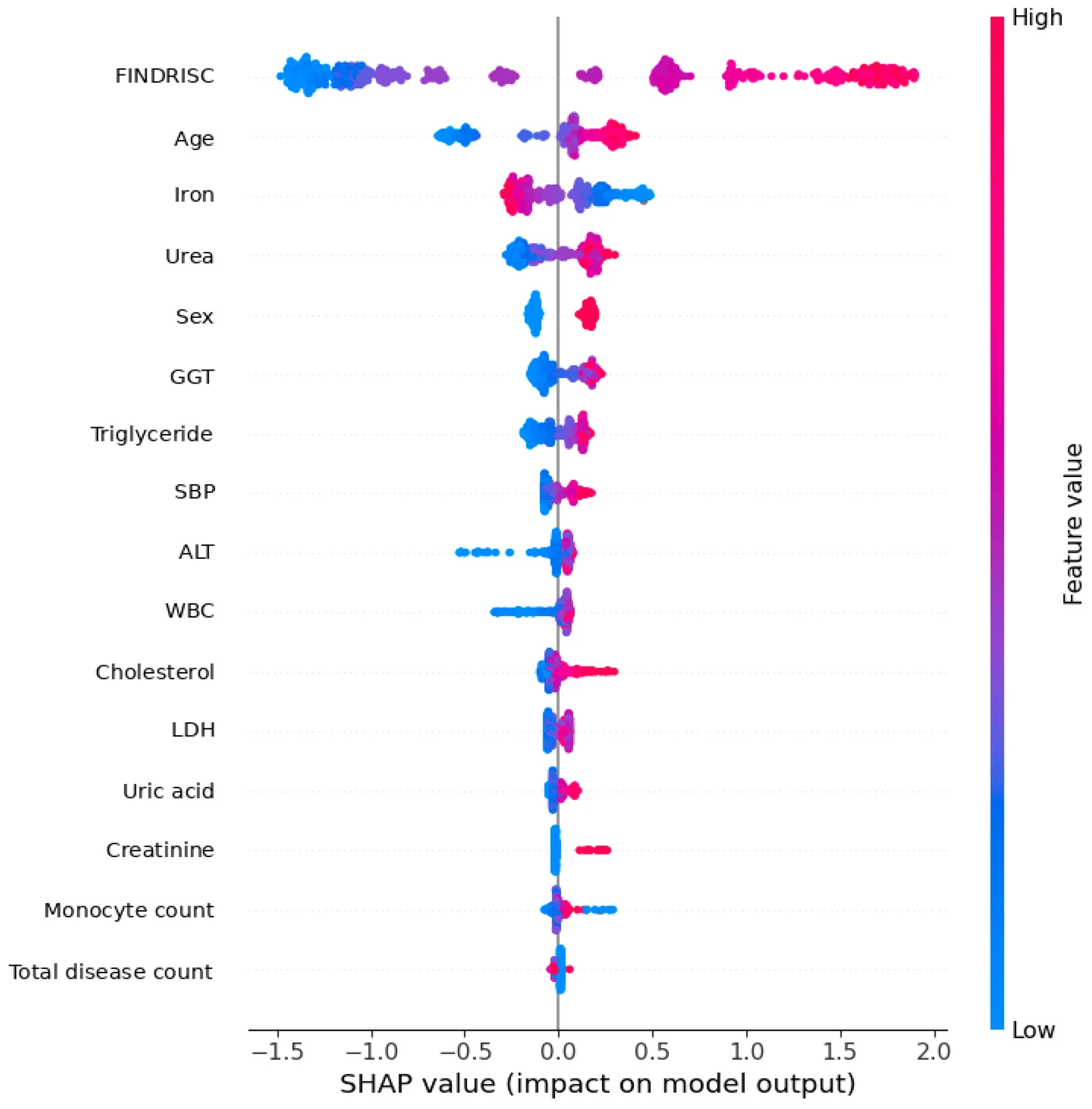
SHAP summary plot for the With FINDRISC CatBoost configuration, showing 15 selected additional variables plus the total FINDRISC (16 displayed features). SHAP values summarize global feature contributions within the CatBoost explanation framework. Abbreviations: FINDRISC, Finnish Diabetes Risk Score; SBP, systolic blood pressure; GGT, gamma-glutamyl transferase; ALT, alanine aminotransferase; WBC, white blood cell count; LDH, lactate dehydrogenase.

**Figure 3:**
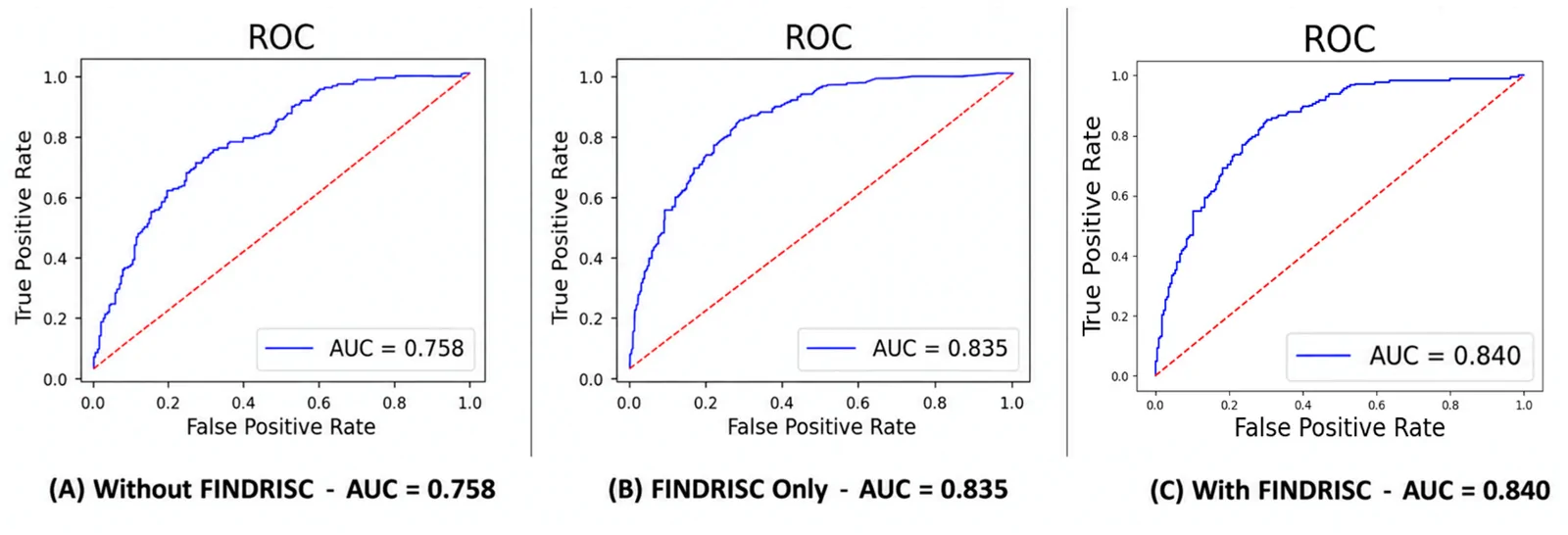
Receiver operating characteristic (ROC) curves on the held-out internal test set (n = 407) for the three CatBoost feature-set configurations: (**A**) Without FINDRISC (AUC = 0.758), (**B**) FINDRISC Only (AUC = 0.835), and (**C**) With FINDRISC (AUC = 0.840). The red dashed diagonal line represents the no-discrimination reference line (AUC = 0.50).

**Table 1:** Baseline characteristics of the analytic study stratified by glycemic outcome.

Characteristic	Normal Glycemia (n = 1098)	Previously Unrecognized Dysglycemia (n = 935)
Age, years	40.65 ± 15.10	54.18 ± 14.53
Male sex, n (%)	473 (43.1%)	465 (49.7%)
FINDRISC	8.60 ± 5.15	16.01 ± 5.26
Systolic blood pressure, mmHg	113.15 ± 19.37	126.07 ± 21.30
Iron, µg/dL	86.90 ± 43.25	80.23 ± 36.11
Total cholesterol, mg/dL	184.24 ± 39.67	201.56 ± 42.65
Triglycerides, mg/dL	123.33 ± 78.55	156.23 ± 99.67
ALT, U/L	20.79 ± 13.22	23.81 ± 18.34
GGT, U/L	20.45 ± 24.05	26.06 ± 25.57
LDH, U/L	190.09 ± 42.46	202.26 ± 48.20
Urea, mg/dL	26.72 ± 8.97	31.09 ± 10.73
Creatinine, mg/dL	0.75 ± 0.22	0.80 ± 0.23
Uric acid, mg/dL	4.91 ± 1.31	5.39 ± 1.41
Monocyte, K/uL	0.54 ± 0.18	0.55 ± 0.19
WBC, K/uL	7.03 ± 1.95	7.26 ± 2.18
Total disease count	0.50 ± 0.91	1.14 ± 1.30

Abbreviations: ALT, alanine aminotransferase; FINDRISC, Finnish Diabetes Risk Score; GGT, gamma-glutamyl transferase; LDH, lactate dehydrogenase; WBC, white blood cell count.

**Table 2:** Five-fold cross-validation average performance metrics.

Feature Set	AUC	Sensitivity	Specificity	Precision	Accuracy	F1-Score
Without FINDRISC	0.78	0.76	0.68	0.67	0.72	0.71
FINDRISC Only	0.84	0.77	0.79	0.76	0.78	0.77
With FINDRISC	0.86	0.80	0.78	0.75	0.79	0.78

Abbreviations: area under the curve (AUC). Without FINDRISC features: iron; systolic blood pressure (SBP); uric acid; triglyceride; sex; lactate dehydrogenase (LDH); white blood cell count (WBC); total disease count; urea; creatinine; monocyte count; alanine aminotransferase (ALT); age; cholesterol; gamma-glutamyl transferase (GGT). FINDRISC, the Finnish Diabetes Risk Score.

**Table 3:** Test set performance metrics with 95% confidence interval for AUC.

Feature Set	AUC (95% CI)	Sensitivity	Specificity	Precision	Accuracy	F1-Score	AP	MCC
Without FINDRISC	0.758 (0.71–0.80)	0.76	0.60	0.61	0.68	0.68	0.698	0.369
FINDRISC Only	0.835 (0.80–0.87)	0.78	0.75	0.71	0.76	0.75	0.784	0.524
With FINDRISC	0.840 (0.80–0.88)	0.80	0.73	0.71	0.76	0.75	0.792	0.527

Footnotes: Binary classification metrics use a probability threshold of 0.50. The paired-bootstrap comparison used 10,000 outcome-stratified resamples of the same held-out test participants. Abbreviations: AP, average precision; AUC, area under the receiver operating characteristic curve; MCC, Matthews correlation coefficient; FINDRISC, Finnish Diabetes Risk Score.

**Table 4:** Confusion matrices for the three CatBoost feature-set configurations on the internal held-out test set at the prespecified 0.50 probability threshold.

Feature Set	True Negative	False Positive	False Negative	True Positive
Without FINDRISC	136	89	43	139
FINDRISC Only	168	57	40	142
With FINDRISC	165	60	37	145

Abbreviations: FINDRISC, Finnish Diabetes Risk Score.

## Data Availability

The data presented in this study are available upon reasonable request from the corresponding author. The data are not publicly available due to privacy and ethical restrictions.

## Supplementary Materials

The following supporting information can be downloaded at https://www.mdpi.com/article/10.3390/healthcare14193214/s1. File S1: TRIPOD+AI checklist. Ref. [13] is cited in Supplementary Materials.

## Abbreviations

The following abbreviations are used in this manuscript:

ADA	American Diabetes Association
AI	Artificial Intelligence
ALT	Alanine Aminotransferase
AP	average precision
AUC	Area Under the Receiver Operating Characteristic Curve
CI	Confidence Interval
FINDRISC	Finnish Diabetes Risk Score
FPG	Fasting Plasma Glucose
GGT	Gamma-Glutamyl Transferase
HbA1c	Glycated Hemoglobin
IDF	International Diabetes Federation
LDH	Lactate Dehydrogenase
MCC	Matthews Correlation Coefficient
ML	Machine Learning
ROC	Receiver Operating Characteristic
SBP	Systolic Blood Pressure
SD	Standard Deviation
SHAP	SHapley Additive exPlanations
T2DM	Type 2 Diabetes Mellitus
WBC	White Blood Cell
XAI	Explainable Artificial Intelligence
